# Should iodine supplementation be universally recommended for pregnant women in Brazil? A position statement from the Thyroid Department of the Brazilian Society of Endocrinology and Metabolism (SBEM)

**DOI:** 10.20945/2359-4292-2025-0170

**Published:** 2025-08-25

**Authors:** Patrícia de Fátima dos Santos Teixeira, Célia Regina Nogueira, Cleo Otaviano Mesa Junior, Helton Estrela Ramos, Léa Maria Zanini Maciel, Mariana de Souza Macedo, Nathalie Silva de Morais, Rosalia do Prado Padovani, Rosalinda Yossie Asato de Camargo, Suemi Marui

**Affiliations:** 1 Programa de Pós-graduação em Endocrinologia, Universidade Federal do Rio de Janeiro, RJ, Brasil; 2 Serviço de Endocrinologia, Hospital Universitário Clementino Fraga Filho, Rio de Janeiro, RJ, Brasil; 3 Departamento de Clínica Médica, Disciplina de Endocrinologia, Faculdade de Medicina, Universidade Estadual Paulista, Botucatu, SP Brasil; 4 Serviço de Endocrinologia e Metabolismo, Hospital de Clínicas, Universidade Federal do Paraná, Curitiba, PR, Brasil; 5 Pontifícia Universidade Católica do Paraná, Curitiba, PR, Brasil; 6 Departamento de Biorregulação, Instituto de Ciências e Saúde, Universidade Federal da Bahia, Salvador, BA, Brasil; 7 Faculdade de Medicina de Ribeirão Preto, Universidade de São Paulo, Ribeirão Preto, SP, Brasil; 8 Departamento de Nutrição, Faculdade de Ciências Biológicas e da Saúde, Universidade Federal dos Vales do Jequitinhonha e Mucuri, Diamantina, MG, Brasil; 9 Programa de Pós-graduação em Ciências da Nutrição, Universidade Federal dos Vales do Jequitinhonha e Mucuri, Diamantina, MG, Brasil; 10 Division of Endocrinology, Diabetes and Hypertension, Brigham and Women's Hospital, Harvard Medical School, Boston, USA; 11 Faculdade de Ciências Médicas, Santa Casa de Misericórdia de São Paulo, SP, São Paulo, Brasil; 12 Unidade de Tireoide, Hospital das Clínicas, Faculdade de Medicina, Universidade de São Paulo, São Paulo, SP Brasil

**Keywords:** Iodine, pregnancy, hypothyroidism

## Abstract

**Background:**

A U-shaped relationship exists between maternal urinary iodine concentration (UIC) and the risk of thyroid dysfunction, adverse pregnancy outcomes, and neurological deficits in offspring. Both iodine deficiency and excess should be avoided during pregnancy. The WHO recommends increased iodine intake during pregnancy due to elevated thyroid hormone production and fetal iodine transfer. In countries with universal salt iodization, additional supplementation is generally not advised, although iodization alone may be insufficient. In Brazil, salt iodization has reduced iodine deficiency disorders, but in 2013, regulatory agencies lowered iodine levels in salt due to high population-wide salt intake. Without national surveys, it remains unclear whether current iodine levels in table salt are sufficient for pregnant women.

**Materials and methods:**

The clinical questions addressed in this document were derived from stakeholder feedback and input from panel members. The group synthesized the available knowledge on this topic by conducting electronic database searches, reviewing and selecting relevant citations, and critically appraising selected studies.

**Results:**

The group recommends exclusive use of regulated iodized salt during pregnancy. Iodine supplementation should be individualized for at-risk pregnant women, including those with chronic gastrointestinal disorders, restricted diets, or malabsorption conditions. Excess iodine intake should be avoided. In alignment with public policies under PNAISAL, health education on appropriate salt use and storage should be reinforced in primary care. Urinary iodine tests should be used for population-level assessment only.

**Conclusion:**

These recommendations aim to support clinical decision-making regarding iodine supplementation during pregnancy in Brazil, thereby improving maternal and fetal health outcomes.

## INTRODUCTION

Iodine is essential for synthesizing thyroid hormones, supporting cell growth, regulating metabolism, and ensuring proper fetal neurological development (^1^,^2^,^3^). Insufficient iodine intake can lead to iodine deficiency disorders (IDDs) (^1^,^2^,^3^,^4^,^5^,^6^), while excessive intake (above 300 µg/day) may result in thyroid dysfunction and increase the risk of thyroid nodules and autoimmune thyroiditis (^7^,^8^,^9^,^10^). The World Health Organization (WHO) and the International Council for Control of Iodine Deficiency Disorders (ICCIDD) provide iodine intake recommendations based on age and physiological status, as shown in Table 1 (^11^). Pregnant women require more iodine due to the stimulation of the hypothalamic-pituitary-thyroid axis, transfer of iodine to the fetus, and increased maternal renal clearance (^11^,^12^,^13^,^ 14^). Lactating women also have higher iodine needs due to iodine excretion through breast milk (^15^).

**Table 1:** Daily iodine dietary intake recommendations for different population groups according to the World Health Organization (WHO) and the International Council for Control of Iodine Deficiency Disorders (ICCIDD) and classification of iodine status according to median urinary iodine concentration in populations of pregnant women

Population group	Recommended intake (µg/day)	Median urinary iodine concentration (µg/L) to classify iodine status			
		Insufficient	Adequate	More than adequate	Excessive
Children aged 0-5 years	90				
Children aged 6-12 years	120				
Adults aged > 12 years	150				
Lactating women	250				
Pregnant women	250	<150	150-249	250-499	≥ 500 µg/L

For pregnant women, achieving an adequate iodine intake (150 µg/day) before pregnancy is ideal. This should increase to 250 µg/day once pregnancy is confirmed and continue during lactation (^11^). In countries with ineffective salt iodization programs, iodine supplementation during pregnancy is recommended (^15^,^16^). However, the WHO advises against universal iodine supplementation in countries with effective salt iodization (^11^), and recent studies support this stance (^17^,^18^). Despite progress in public health policies, some studies suggest that iodized salt alone may not provide adequate iodine intake for all pregnant women (^18^,^19^,^20^,^21^). A systematic review of 61 studies including 163,021 pregnant women found that 53% had insufficient iodine intake (^22^).

The American Thyroid Association (ATA) (^16^) and European Thyroid Association (ETA) (^23^) recommend daily iodine supplementation of 150 µg throughout pregnancy in their most recent guidelines. However, this recommendation is not universally adopted (^24^). Iodine deficiency risk is particularly high in women living in iodine-deficient regions or those following restrictive diets (such as vegan or non-dairy diets or using non-iodized salt) (^25^,^26^). Early iodine supplementation, especially starting 3 months before conception, is associated with greater benefits (^16^).

In Brazil, iodine fortification of table salt is mandatory by law, reducing the risk of IDDs. However, concerns have arisen after the regulatory reduction of iodine content in table salt from 20-60 mg/kg to 1545 mg/kg in 2013 (^27^). This decision aimed to address high salt consumption, but there are concerns about insufficient iodine levels in populations with restricted salt intake or specific diets (^27^).

The iodine status of pregnant women in Brazil has been assessed through regional studies (Table 2), since no national data have been published yet (^28^,^29^,^30^,^31^,^32^,^33^,^34^,^35^,^36^,^37^,^38^,^39^,^40^). There is growing interest in identifying subgroups of pregnant women at risk of insufficient or excessive iodine intake. This Position Statement, developed by specialists, aims to provide insights into iodine supplementation recommendations for pregnant women in Brazil and identify gaps in research for future studies and public policies to ensure the health and safety of this vulnerable population.

**Table 2:** Iodine status of populations of pregnant women in Brazil according to regional studies

Study	Region (City, State)	N	Demographic characteristics	UIC (µg/L)	Prevalence of different iodine status	
					Insufficient	Excessive (UIC > 500 µg/L)
Ferreira, 2014	Southeast (Ribeirão Preto, SP)	191	- Recruitment before 2013 in primary care units of a city in the interior of São Paulo - Median age 25 (18-42) years - 20.9% smokers - 24% with a history of miscarriage	137.7	57%	None (but 9.9% with UIC > 250 µg/L)
Mioto, 2018 (2012-2016)	Southeast (São Paulo, SP)	273	- University hospital and a prenatal project program in a community in the city of São Paulo - Mean age 27.7 ± 6.5 years - All trimesters - Exclusion criteria: high-risk pregnancies (kidney disorders, hypertension, diabetes and HIV-positive), preeclampsia, and known thyroid disease (including those with TSH level > 10 mlU/L).	144	52.2%	None (but 4.4% with UIC > 250 µg/L)
Saraiva, 2018	Southeast (Rio de Janeiro, RJ)	244	- Primary care (public health) - Pregnant women from a coastal city - First trimester - Mean age 26.5 ± 5.5 years	221	48%	4.5%
Candido, 2024	All regions -EMDI/Brazil (11 municipalities in nine states and the Federal District)	1891	- Pregnant women attending the public health system and residents in urban and rural areas of each municipality. - 45.6% with overweight	186.6	37.6%	3.6% (but 28.7% > 250 µg/L)
Momentti, 2023	Southeast (Ribeirão Preto, SP)	266	- Two health care units - Any gestational age (those in the second trimester had median UIC compatible with iodine insufficiency)	180	38%	27.8% (UIC >250 µg/L*)
Scherr, 2022	Southeast (Belo Horizonte, MG)	30	- Primary health care units - Mean age 26.7 ± 5.8 years - 28.3% overweight and 24.5% obese - 7.5% smokers	216.7		
Rates, 2021	Southeast (Vespasiano, MG)	69 (pregnant teenagers)	- Normal-risk prenatal care - Interior city from a non-coastal region - 43.3% overweight - Median age 27 (18-42) years - Only one smoker - 50% nulliparous and in first pregnancy - No participants obesity	108.2 (mean)	71%	None
Macedo, 2017	Southeast (Diamantina, MG)	209	- Only third trimester - Public primary care system	94.6	72%	0.5% (but 8.4% > 250 µg/L)
Felchner, 2023	South (Curitiba, PR)	225	- Only first trimester - Included 10 health care district systems in Curitiba	158.2	47%	2.6% (but 20.9% > 250 µg/L)
Sant'Ana Leone de Souza, 2020	North East (Salvador, BH)	241 (high-risk pregnancies)	- High-risk pregnancies - High blood pressure and salt-restrict diet associated with low UIC	119	61.8%	-
Marchi Junior, 2023	Southeast (Botucatu, SP)	25	- Prenatal care units in the public health care system - Mean age 27.8 ± 10.8 years	231.4 (mean)	-	47.9% (> 250 µg/L*)

* The category of excess iodine was analyzed along with the category > 250.

## WHAT IS THE BEST WAY TO DEFINE THE IODINE STATUS IN A GROUP OF PREGNANT WOMEN?

Iodine is excreted in urine; therefore, urinary levels of this micronutrient are directly related to an individual's iodine intake (^4^).

To assess the iodine status of a population, two indicators are considered: clinical and biochemical. Clinical indicators of iodine insufficiency are related to the presence of goiter and the detection of overt or subclinical hypothyroidism (^11^). The urinary iodine concentration (UIC) serves as the most widely used biochemical marker for assessing iodine sufficiency in a population and correlates positively with iodine intake (^4^). Beyond its diagnostic value, UIC measurement is an efficient, inexpensive, innocuous, and technically easier method compared with other tests used to determine a population's iodine status.

Monitoring programs for iodine sufficiency in the general population rely on median UIC data collected from schoolchildren aged 6-12 years and extrapolate these results to the whole population. However, this demographic, along with non-pregnant women, does not provide an adequate sample for assessing iodine sufficiency in pregnant women, whose iodine dietary requirements increase significantly during pregnancy (^11^).

Notably, UIC correlates with the severity of disorders associated with iodine deficiency and helps determine the urgency required for corrective measures (^11^). For the general population, adequate iodine intake is defined as a median UIC > 100 µg/L among schoolchildren from that population, with < 20% of the schoolchildren having a UIC < 50 µg/L, while moderate and severe iodine deficiencies in the population are defined by a median UIC 20-49 µg/L and < 20 µg/L, respectively, in their schoolchildren (^4^). For pregnant women, the iodine status levels according to UIC are shown in Table 1.

While UIC is useful for monitoring a population's iodine status, it has limited value in assessing individual iodine intake due to significant variations throughout the day and between days (^41^). Therefore, UIC measurement should not be used in clinical practice to assess iodine sufficiency in individual patients.

For epidemiological purposes, median UIC values should be considered instead of mean values, as the inclusion of a few UIC samples with exceptionally high or low values can considerably skew the mean (^5^,^42^,^43^).

There are two primary methods for collecting urine for UIC measurement: spot (random) urine samples and 24-hour urine samples. Random urine samples may be affected by urine volume and hydration status, leading to inaccurate estimates of iodine deficiency prevalence (^5^). In contrast, 24-hour urine samples are more accurate for assessing iodine excretion and intake, as they directly reflect diet. However, due to difficulties in collecting 24-hour samples, population studies often use median values from random urine samples, as recommended by the WHO (^43^).

Various techniques exist for measuring UIC (^44^). In Brazil, the modified Sandell-Kolthoff method is commonly used, while inductively coupled plasma mass spectrometry (ICP-MS) is considered the most accurate. Notably, UIC may also be expressed relative to urinary creatinine to account for fluid intake variations.

Thyroglobulin, a protein secreted by the thyroid gland, is an indicator of thyroid function and iodine deficiency, particularly when UIC is below 100 µg/L (^18^). It correlates with thyroid volume (^45^,^46^,^47^) and is useful for screening congenital hypothyroidism in infants. Thyroglobulin can be measured using filter paper blood samples, which may also be applied for screening pregnant women for iodine deficiency. However, its reliability may be compromised in individuals with antithyroglobulin antibodies (^45^,^46^,^47^).

## WHAT DATA EXIST REGARDING THE IODINE STATUS OF PREGNANT WOMEN IN BRAZIL?

Historically, Brazil's iodine sufficiency monitoring has focused on periodic surveys of schoolchildren, and not on pregnant women. The most recent nationally representative surveys, The National Salt Iodization Impact Assessment Survey (PNAISAL), conducted in 2008-2009 and 2013-2014, collected data from 18,864 schoolchildren aged 6-14 years. A median urinary iodine concentration (UIC) of 276.7 µg/L indicated adequate iodine status, with a notable proportion of subjects showing overconsumption (^48^). However, these data do not represent all at-risk groups and are outdated, as UIC reflects recent iodine intake.

In contrast, local studies on pregnant women have shown mixed results regarding iodine nutritional status, revealing a heterogeneous landscape with varying levels of deficiency, adequacy, and, to a lesser extent, excess iodine intake (Table 2). This highlights the need to update policies on iodine deficiency disorders (IDDs), particularly by including pregnant women in monitoring efforts and periodic surveys.

In addition to improving population monitoring, it is vital to review the relevance of current indicators used to evaluate Brazil's salt iodization policy. While Brazil's strategy has focused on iodizing salt for domestic consumption, the growing intake of industrially processed and ultra-processed foods affects overall salt intake. Therefore, there is an urgent need to assess the impact of these foods and their salt content on daily iodine intake, particularly among pregnant women, to ensure effective iodine deficiency prevention.

In Ribeirão Preto, located in the interior of São Paulo state, a survey conducted with 191 pregnant women revealed an insufficient iodine status (^32^). A subsequent study with 266 other pregnant women from the same city revealed an adequate median UIC, despite significant variability in the UIC of collected samples; this indicates a heterogeneous epidemiological scenario with persistent rates of iodine deficiency, even after 7 years (^31^).

In the capital city of São Paulo (SP), a study conducted between 2012 and 2016 showed a 52% prevalence of iodine deficiency and a median UIC of 144 µg/L (^33^), indicating insufficiency of this micronutrient. It is important to highlight the specific characteristics of this group of pregnant women, as their prenatal care was performed in a tertiary hospital rather than a primary health unit. Similar findings were reported in a study from Bahia evaluating women with high-risk pregnancies—particularly those with high blood pressure, who are at risk for iodine deficiency (^39^).

Between 2014 and 2017, in the coastal city of Rio de Janeiro, Brazil, 244 healthy pregnant women were evaluated (^28^), with urine samples collected on multiple occasions throughout the first trimester. Despite an adequate median UIC (221 µg/L), it was found that 48.7% of the women had at least one measurement indicating iodine insufficiency, and 4.5% had iodine excess.

In Minas Gerais state, various studies with different sample sizes and methodologies have shown that the median UIC aligns with insufficient iodine status. However, a study (^34^) with a small sample of women attending the Prenatal Outpatient Clinic of the Federal University in the capital city (Belo Horizonte) reported a normal median UIC. Another study conducted in the same state, in the municipality of Diamantina, analyzed the iodine status of 209 pregnant women in their third trimester, who were followed up in the primary health care network of the municipal headquarters, and found a lower median UIC (94.6 µg/L), with approximately 72% of participants presenting UIC < 150 µg/L (^37^). A third study from another countryside town in Minas Gerais (Vespasiano), evaluating 69 pregnant teenagers, also revealed the same scenario (^36^).

The Multicenter Study of Iodine Deficiency (EMDI-Brazil) is the most extensive study assessing the nutritional iodine status of pregnant women in Brazil. It evaluated 1,891 urine samples from pregnant women in all three trimesters in 11 municipalities distributed across five Brazilian macro-regions between 2018 and 2021 (^35^). The median UIC among the pregnant women in this study was adequate (186.6 µg/L), consistent with findings from a smaller study conducted during the same period in southern Brazil, specifically in Paraná (^38^). Notably, there is a significant discrepancy in the prevalence of iodine deficiency across different regions of the country, with rates of 23.5% and 30.1% found in Viçosa and Belo Horizonte (both in Minas Gerais), respectively, compared with 51.4% in Vitória (Espírito Santos) and 62.2% in Palmas (Tocantins).

## WHAT ARE THE SOURCES OF IODINE FOR PREGNANT WOMEN AND WHAT FACTORS INFLUENCE THEIR IODINE STATUS?

Pregnancy requires a healthy diet, and insufficient intake of essential micronutrients like iron, zinc, iodine, copper, and selenium has been linked to complications and adverse neonatal health outcomes, making it a global health concern (^49^,^50^,^51^). While obtaining adequate nutrients through diet is preferred, meeting some pregnancy-specific micronutrient needs can be challenging to achieve with diet alone (^51^).

Iodine is primarily found in eggs, dairy, and seafood, but most foods contain relatively low levels of it. The iodine content in food is influenced by environmental factors, agricultural practices, food preparation methods, and conservation techniques (^52^,^53^). For instance, iodine levels in plant-based foods depend on the iodine concentration in the soil. Coastal regions typically have higher iodine in the soil, while inland areas, particularly at higher altitudes, tend to have iodine-deficient soils, leading to lower iodine in local foods (^53^). Cooking methods can also affect iodine levels; boiling reduces iodine because it is water-soluble, whereas steaming and microwaving preserve more iodine. Some food processing techniques, such as refining grains, also reduce iodine content because the outer layers, which are rich in iodine, are removed (^54^).

Table salt is a major source of iodine, but its iodine content can decline over time when exposed to air and light. Proper storage in airtight, dark containers can help preserve iodine levels, and it is known that salt stored in plastic bags loses iodine more quickly than salt stored in glass containers (^55^,^56^).

**Figure 1:**
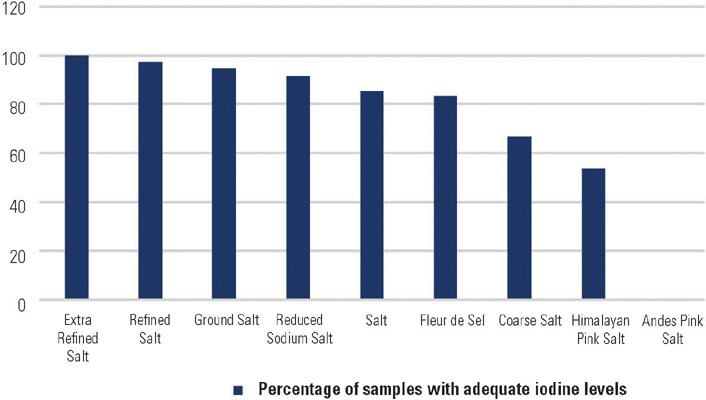
Percentage of samples with adequate iodine levels in salt for human consumption by product type according to the Brazilian National Health Surveillance Agency (Anvisa) 2019 report.

Monitoring policies are important for ensuring adequate iodine levels in table salt. The National Health Surveillance Agency (Anvisa) (^57^) analyzed 742 samples of salt for human consumption collected from 17 Brazilian states (Figure 1). Of these samples, 88.3% fell within the acceptable range (15-45 mg/kg). This is the lowest range reported since 2010. Among 87 samples deemed unsatisfactory, Himalayan pink salt accounted for 62.1% (n = 54). However, in a study conducted in Rio de Janeiro, analyzing table salt samples from pregnant women's homes, less than 2% had iodine concentrations below governmental recommendations (^28^). These findings suggest that the adequacy of public regulation regarding iodine concentrations in table salt may diverge by region in Brazil.

In addition to dietary factors, sociodemographic factors also play a role in determining iodine nutritional status. Factors such as income, education, and housing conditions can influence both the quality and quantity of available food, directly affecting the adequacy of iodine intake (^58^).

To ensure that everyone receives sufficient iodine, both the WHO and the United Nations Children's Fund (Unicef) recommend universal salt iodization as a global strategy (^11^). However, in some countries, salt iodization is only feasible in certain regions. Evidence suggests that in areas where universal salt iodization is not fully implemented, pregnant and lactating women and children under 2 years of age may not receive adequate amounts of iodine (^59^).

In these contexts, depending on the region and the percentage of family members in a given area with access to iodized salt, iodine supplementation may be necessary to ensure that pregnant women receive adequate iodine intake. Both WHO and Unicef recommend iodine supplementation for pregnant and lactating women in countries where less than 20% of families have access to iodized salt, until an appropriate salt iodization program is established. In countries where 20%-90% of households have access to iodized salt, efforts should be made to accelerate salt iodization or to assess the feasibility of increasing iodine intake through supplements or iodine-fortified foods for the most susceptible groups (^43^).

The specific amount of iodine that should be supplemented is a local decision, depending on the extent of existing iodine deprivation (^1^,^60^). Current ATA guidelines recommend starting low-dose iodine supplements, ideally 3 months before pregnancy, for women living in regions with known mild to moderate iodine deficiency (^16^). In light of the available evidence, a feasible approach is to recommend that all pregnant women regularly consume iodized salt in their diet, supplemented by an oral intake of 150 µg of iodine per day in the form of potassium iodide, ideally starting at least 3 months before conception. Special attention should be given to women at higher risk of iodine deficiency, including those with chronic bowel malabsorption disease, celiac disease (^61^), lactose intolerance (^62^), or those following specific dietary regimens such as vegan or low-salt diets (^34^).

It should be noted that iodine in breast milk is more readily absorbed. However, its concentration reflects the mother's diet and nutritional iodine status; if the mother's iodine intake is inadequate, the iodine content in breast milk will also be insufficient. With adequate maternal iodine supplementation, breastfeeding infants should achieve adequate iodine status (^63^,^64^).

## WHAT IS THE EVIDENCE FOR IODINE SUPPLEMENTATION IN PREGNANT POPULATIONS?

Pregnant and lactating women are at increased risk of iodine deficiency due to higher iodine demands, even in countries with effective salt iodization programs (^65^). Women following restrictive diets, such as vegan or dairy-free diets, often fail to meet the recommended iodine intake and require special attention (^26^,^66^). While severe iodine deficiency has become rare due to global IDD programs, mild-to-moderate iodine insufficiency persists in many countries. Iodine supplementation and salt fortification in areas of severe deficiency have reduced cretinism and infant mortality rates (^4^). In cases of mild-to-moderate deficiency, increased thyroid activity compensates for low iodine intake, maintaining normal thyroid hormone levels in most individuals. However, it remains unclear how these adaptations affect pregnancy and the potential risks to both the mother and fetus.

A recent study in an iodine-sufficient region of China assessed 7,190 pregnant women and found a U-shaped relationship between serum TSH levels and urinary iodine concentrations (UICs), with the lowest prevalence of hypothyroidism at UICs of 150-249 µg/L (^67^). Mild-to-moderate iodine deficiency during pregnancy has been linked to negative effects on child neurodevelopment, such as motor skills, language, IQ, and school performance (^68^,^69^,^70^,^71^). However, randomized controlled trials have not consistently shown that iodine supplementation improves children's mental and behavioral development in these cases (^72^).

Variability in study designs and populations could explain the mixed results. Factors such as the degree of iodine deficiency, the timing and dosage of supplementation, and methods used to assess child development contribute to these inconsistencies. The impact of mild iodine deficiency on fetal brain development is subtle, requiring more sensitive tools to assess the effects of iodine supplementation. Additionally, the optimal timing for starting iodine supplementation during pregnancy remains undefined.

An Italian cohort study showed that iodine supplementation (through iodized salt for over 2 years before conception) improved children's IQ scores at ages 6-12 years, suggesting that starting supplementation during pregnancy may be too late to prevent adverse effects (^73^).

International health authorities, including the WHO, recommend 150 µg of iodine daily for pregnant, lactating women, and those planning a pregnancy (^16^,^23^,^74^). However, the WHO advises iodine supplementation only for those in areas with low iodized salt coverage (^13^). While previous studies have shown improvements in some maternal thyroid indexes with iodine supplementation during pregnancy (^75^,^76^), other data have raised concerns about its safety for pregnant women. According to a study by Shi and cols., UICs > 250 µg/L in the first trimester of pregnancy are associated with higher serum TSH levels and an increased risk of subclinical hypothyroidism (SCH). In that population, UICs > 500 µg/L were also associated with isolated hypothyroxinemia (^75^). A cohort study in an iodine-sufficient area of Brazil also found that pregnant women with excessive iodine intake had up to a sixfold greater risk of SCH compared with those with adequate iodine uptake (^28^,^76^).

Additionally, iodine status during pregnancy appears to influence glucose metabolism and insulin secretion. Iodine imbalance during pregnancy - whether due to deficiency or excess - is associated with a higher risk of gestational diabetes mellitus (^77^,^78^). Another area of concern is the potential adverse effects of excessive maternal iodine intake on fetal mental development. In the Norwegian Mother and Child Cohort Study (MoBa), children born to women who received iodine supplementation starting in the first trimester were at a higher risk for a diagnosis of attention deficit hyperactivity disorder (ADHD) and had increased ADHD symptom scores at 8 years of age compared with those born to mothers who did not receive supplementation (^68^). More recent observational studies have also shown that maternal iodine excess in the first trimester of pregnancy can adversely affect infants' neurodevelopment. For example, there is evidence of language developmental delays at 18-24 months (^79^), as well as lower mental and psychomotor development indexes at 2 months (^80^) and 12 months (^3^), even when maternal thyroid function is normal.

## WHAT IS THE STATE OF THE ART IN BRAZIL REGARDING A POSSIBLE POSITION STATEMENT ON IODINE SUPPLEMENTATION FOR PREGNANT WOMEN?

In Brazil, Anvisa has established through Collegiate Board Resolution No. 23 dated April 24, 2013, that salt marketed for human consumption must contain 15-45 mg/kg of iodine (^65^). However, according to the 2019 Report Monitoring on the Iodization of Salt Intended for Human Consumption, this percentage represented the lowest in recent years (^57^). Currently, there are no positions from the Ministry of Health or from Anvisa regarding iodine supplementation for pregnant women. The National Program for the Prevention and Control of Iodine Deficiency

Disorders *(Pró-Iodo),* coordinated by the Ministry of Health in partnership with several agencies and entities, has the following main lines of action to promote the elimination of iodine deficiency in Brazil:

- Monitoring the iodine content of salt for human consumption.
- Tracking the health impact of salt iodization in the population.
- Updating the legal parameters for the iodine content of salt intended for human consumption.
- Implementing information, education, communication, and social mobilization strategies continuously.

Despite these efforts, there is no specific recommendation for pregnant women, including those at higher risk for iodine deficiency, though the consequences of deficiency are recognized. The only existing guidelines are those provided in primary health care for the general population and include advising against consuming salt intended for livestock and other animals, as it has a much lower iodine concentration per kilogram than required for humans. Additionally, guidelines emphasize the correct purchase and storage of table salt, ensuring the quality of the existing iodine is maintained.

## WHAT INITIATIVES SHOULD BE TAKEN TO ADDRESS THIS PROBLEM AND TRANSLATE EVIDENCE INTO CLINICAL PRACTICE?

The authors of this Position Statement recommend measures outlined in Table 3 to guide iodine supplementation decisions for pregnant women in Brazil. They also propose conducting national or subnational surveys to assess iodine intake through food frequency questionnaires or 24-hour food recall, considering iodine-rich foods like eggs, dairy, fish, and seafood, along with iodized salt. These surveys would help identify pregnant women at risk of deficiency and allow for the assessment of their UIC.

**Table 3:** Recommendations for iodine supplementation of pregnant women in Brazil

Expert Opinion #1	A U-shaped relationship likely exists between maternal urinary iodine concentration (UIC) and the risk of maternal thyroid dysfunction, adverse pregnancy, and children's neurological outcomes. Therefore, both iodine insufficiency and excess should be avoided in pregnancy
Expert Opinion #2	There is a consensus on the importance of preventing severe iodine deficiency during pregnancy. Currently, there is no evidence indicating that pregnant women in Brazil are at risk for severe iodine insufficiency. However, mild-to-moderate iodine insufficiency has been observed among certain subgroups of pregnant women in Brazil despite the successful implementation of a universal salt iodization program, based on data from subnational surveys.
Expert Opinion #3	It is essential to closely monitor iodine status in vulnerable populations, especially pregnant women, to prevent the adverse consequences of iodine imbalances for both the mother and the fetus. It is urgent to include pregnant women in the systematic monitoring of iodine status in nationwide surveys.
Expert Opinion #4	Iodine supplementation can be considered for pregnant women at increased risk of iodine deficiency, such as those on restrictive diets (vegan and non-dairy diets and diets without iodized salt) or with malabsorption conditions. For these women in particular, we recommend a daily oral supplement containing a maximum of 100-150 µg of iodine in the form of potassium iodide (to avoid excessive supplementation), which should ideally be started 3 months before planned pregnancy.
Expert Opinion #5	Pregnant women or women planning to become pregnant should use table salt that is produced and distributed in Brazil. All salt for human consumption produced in Brazil is subject to regulatory policies that ensure the addition of 15-45 mg of iodine per kg of salt. This recommendation should begin 3 months before planned pregnancy and continue throughout gestation.
Expert Opinion #6	Measuring individual iodine levels through urinary iodine excretion is not a reliable marker of individual iodine status and should not be incorporated into clinical practice.
Expert Opinion #7	National educational policies regarding the storage and consumption of iodized salt, as outlined in the National Salt Iodization Impact Assessment Survey (PNAISAL) report, should be widely disseminated, especially in primary health care units.

Since 90% of ingested iodine is excreted in urine, UIC is commonly used to assess dietary iodine sufficiency. However, UIC may not accurately reflect iodine intake in pregnant women due to iodine transfer to the fetus and potential storage in the placenta (^72^).

Additionally, Brazil's Surveillance System of Risk and Protective Factors for Chronic Diseases by Telephone Survey (Vigitel) conducts annual interviews on food consumption and other factors. This system, which surveys individuals in the capitals of all Brazilian states, could serve as a model for a national survey on iodine intake (^81^).
